# RXLR-Mediated Delivery of Exogenous CRr Protein Reduces Clubroot in Tumorous Stem Mustard and Reshapes the Rhizosphere Microbiome

**DOI:** 10.3390/plants15213373

**Published:** 2026-11-01

**Authors:** Diandong Wang, Jingjing Liao, Tailin Chen, Zhaoming Cai, Zhiyi Chen, Qing Wang

**Affiliations:** College of Modern Agriculture and Bioengineering, Yangtze Normal University, Chongqing 408000, China; ddwangwill@163.com (D.W.); 10091123@163.com (J.L.); 17830789596@163.com (T.C.); caizhaoming-2000@163.com (Z.C.); 13983192099@163.com (Z.C.)

**Keywords:** RXLR-fused CRr protein, clubroot, *Plasmodiophora brassicae*, rhizosphere microbiome, metabolic reprogramming, *Brassica juncea* var. tumida

## Abstract

Clubroot, incited by the obligate biotrophic protist *Plasmodiophora brassicae*, poses a severe threat to cruciferous crop production worldwide. Building on the concept of exogenous NLR protein delivery, we tested whether a bacterially expressed CRr protein (a recombinant Crr1a protein fused with an RXLR motif) could confer protection against clubroot in tumorous stem mustard (*Brassica juncea* var. tumida). Seedling roots were exposed to a crude lysate harboring the RXLR–*Crr1a* chimera, after which the fate of the protein and its downstream consequences were monitored across molecular, ecological, and metabolic levels. Time-resolved fluorescence imaging demonstrated that the recombinant signal became broadly distributed throughout the root system within a 24 h window, concomitant with a marked alleviation of disease severity. An empty vector control (equal protein, pET-28a(+) lysate) showed no significant protection versus the pathogen-only control (*p* > 0.05), suggesting that the observed protection is associated with the CRr protein lysate rather than general bacterial components. At the microbial level, the treatment fostered a more interconnected rhizosphere co-occurrence network and selectively promoted taxa, recognized as beneficial bacteria. At the metabolic level, several defense-associated compounds were elevated, among them jasmonic acid methyl ester, (2R)-2-hydroxyphenethylglucosinolate, 8-hydroxyluteolin 4′-methyl ether 8-glucuronide, and 4-hydroxyglucobrassicin. Furthermore, soil populations of beneficial bacteria including the Gram-positive genera *Bacillus* and *Priestia* exhibited strong positive associations with host defense metabolites. Collectively, these findings support a model in which externally supplied CRr protein lysate induces a response that can be observed at the metabolite and microbiome levels via rapid protein dissemination. This work lays a conceptual foundation for protein-based, microbiome-oriented management of soil-borne diseases without genetic modification of the crop.

## 1. Introduction

The soil-borne protist *Plasmodiophora brassicae*, an obligate biotrophic pathogen, causes clubroot disease—a condition marked by severe root swelling and overgrowth. This physiological damage severely weakens nutrient and water uptake in host plants, leading to major yield losses. *P. brassicae* infects a broad range of Brassica species across diverse crop types. This includes cornerstone crops such as rapeseed (*Brassica napus*), Chinese cabbage (*B. rapa* sub sp. pekinensis), bok choy (*B. rapa* subsp. chinensis), kale (*B. oleracea* var. acephala), cauliflower (*B. oleracea* var. botrytis), and tumorous stem mustard (*B. juncea* var. tumida) [1]. In key cultivation hubs for tumorous stem mustard (e.g., Fuling, Chongqing), infection rates result in production losses exceeding 60% [2], posing a serious threat to the region’s agricultural sustainability.

To date, multiple major clubroot resistance genes have been identified in Chinese cabbage (*B. rapa*). Key reported loci include *CRa* [3], *CRb* [4], *CRc*, *CRk* [5], *Crr1a*, *Crr2* [6], *Crr3* and *Crr4* [7]. Genetic mapping has localized these genes to specific linkage groups: *Crr1a* on A8, *Crr2* on A1, *Crr4* on A6, *CRc* on A2, and *Crr3*, *CRa*, *CRb*, and *CRk* on A3 [8]. Functional characterization studies have demonstrated that ectopic overexpression of *Crr1a* in Arabidopsis or Chinese cabbage significantly reduces clubroot incidence in T1 generation plants. Furthermore, the introgression of *CRb* candidate genes into canola (*B. napus*) revealed that the overexpression of Bra012540 and Bra012541 confers partial resistance to *P. brassicae* [9].

Using genetic resistance remains the most sustainable and affordable way to manage clubroot. The landmark cloning of *Crr1a* revealed it to be a typical TIR-NB-LRR receptor conferring race-specific immunity against *P. brassicae* [9]. Still, using this trait widely is limited by the long process of introgression breeding, linkage drag, and crossing barriers. While precision breeding through transgenics or genome editing provides a direct way to obtain functional alleles, it is still held back by strict regulations, public doubts, and limited genetic scope [10]. These limits call for a major change: supplying working resistance proteins from outside the plant, instead of changing the genome. Recent progress in NLR engineering—especially changes to LRR domains and building chimeric scaffolds—points to the promise of protein-based immunity [11]. A key part of this approach is overcoming the problem of ensuring that proteins reach plant cells: adding an RXLR motif allows the CRr protein (a recombinant *Crr1a* protein fused with an RXLR motif) to enter the cell cytoplasm and turn on the plant’s own immune pathways without genetic modification [12]. This external activation is associated with changes in root exudate composition, which may act as a signal for reshaping rhizosphere microbial communities.

Given that root exudates are primary determinants of microbial assembly [13,14] and that NLR-mediated signaling (e.g., via salicylic acid/jasmonic acid) can recruit beneficial microbiota [15,16], we hypothesized that externally applied CRr protein could produce the same effects. While microbiome-driven disease suppression has been reported in banana-Fusarium systems and other Brassica-clubroot studies [17], triggering this effect with external protein in tumorous stem mustard has not been tested yet. Here, we combine amplicon sequencing and metabolomics to figure out how applying CRr protein changes rhizosphere bacterial communities and root metabolites, aiming to build a working model connecting protein-based immunity to microbiome-driven clubroot resistance in *B. juncea* var. tumida.

## 2. Results

### 2.1. Movement of Recombinant Protein in Tumorous Stem Mustard Roots

**Figure 1:**
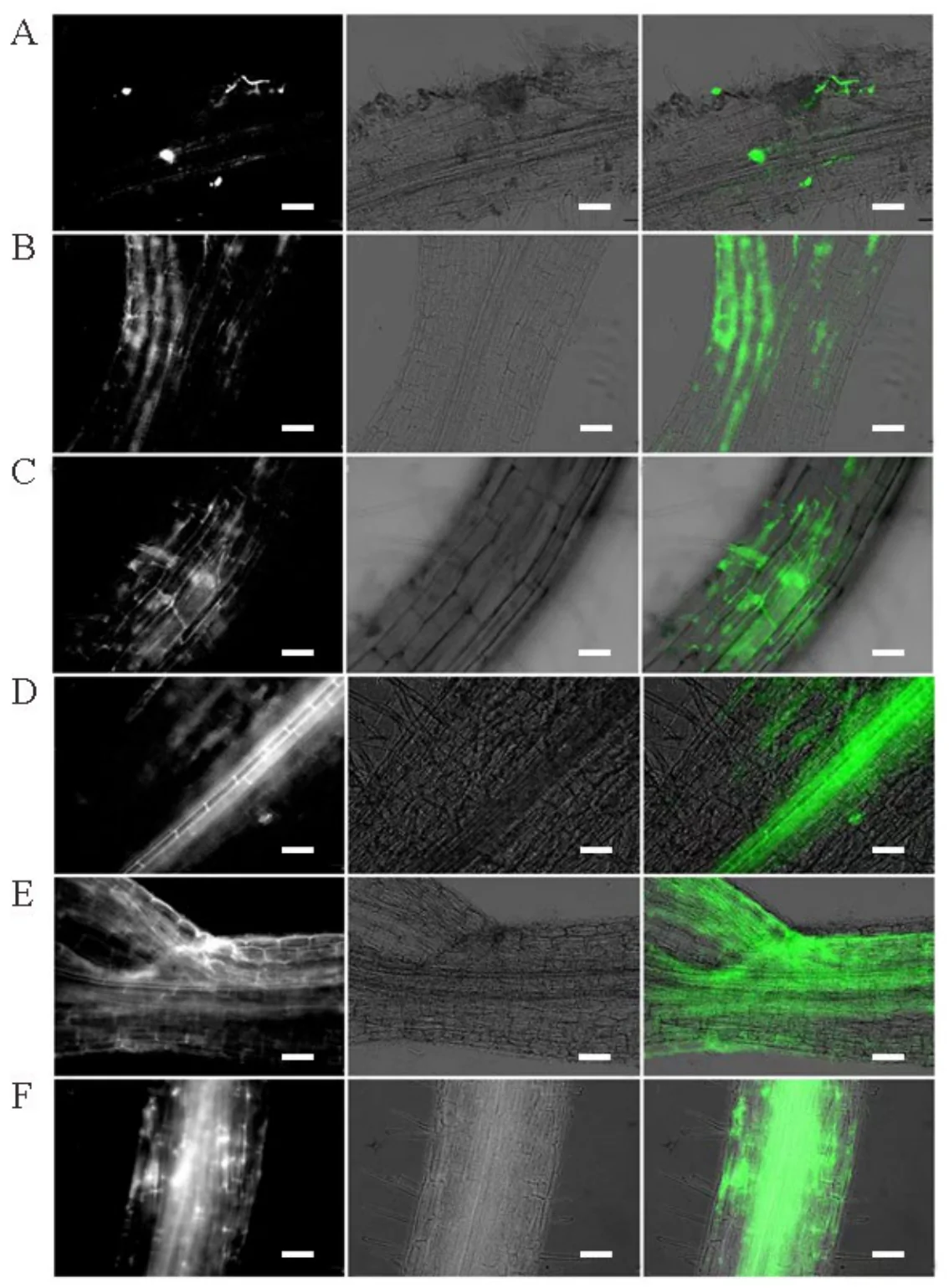
Time-resolved distribution of the recombinant CRr protein within tumorous stem mustard roots. (**A**) 1 hpi, earliest detectable fluorescence confined to the root cortex; (**B**) 3 hpi, radial expansion of the signal across cortical cell layers; (**C**) 6 hpi, initial penetration into ground tissues; (**D**) 9 hpi, widespread signal in inner tissues; (**E**) 12 hpi, fluorescence concentrated in the stele and vascular region; (**F**) 24 hpi, complete tissue saturation indicating systemic distribution. From left to right: bright field, fluorescence channel, and merged overlay. Scale bars: 20 μm.

Time-lapse fluorescence imaging captured the spatiotemporal dynamics of protein entry into the root system. Discrete fluorescent foci emerged in the root cortex as early as 1 hpi (Figure 1A), followed by lateral signal propagation across the cortical layer by 3 hpi (Figure 1B). Initial incursion into the ground tissue was evident at 6 hpi (Figure 1C), transitioning to pervasive movement of inner root tissues by 9 hpi (Figure 1D). Characteristic fluorescence subsequently arose within the stele and vascular bundles at 12 hpi (Figure 1E), ultimately achieving full-tissue saturation by 24 hpi (Figure 1F). These data confirm that the fusion protein establishes systemic movement throughout tumorous stem mustard roots within a 24 h window. While fluorescence tracking confirmed the systemic translocation of the fusion protein throughout root tissues within 24 h, these images do not by themselves establish cytoplasmic delivery. Accordingly, to guarantee comprehensive dissemination of the protein payload prior to pathogen challenge, inoculations with *P. brassicae* resting spores were performed 24 h following protein treatment.

### 2.2. Control Efficacy Against Clubroot

**Figure 2:**
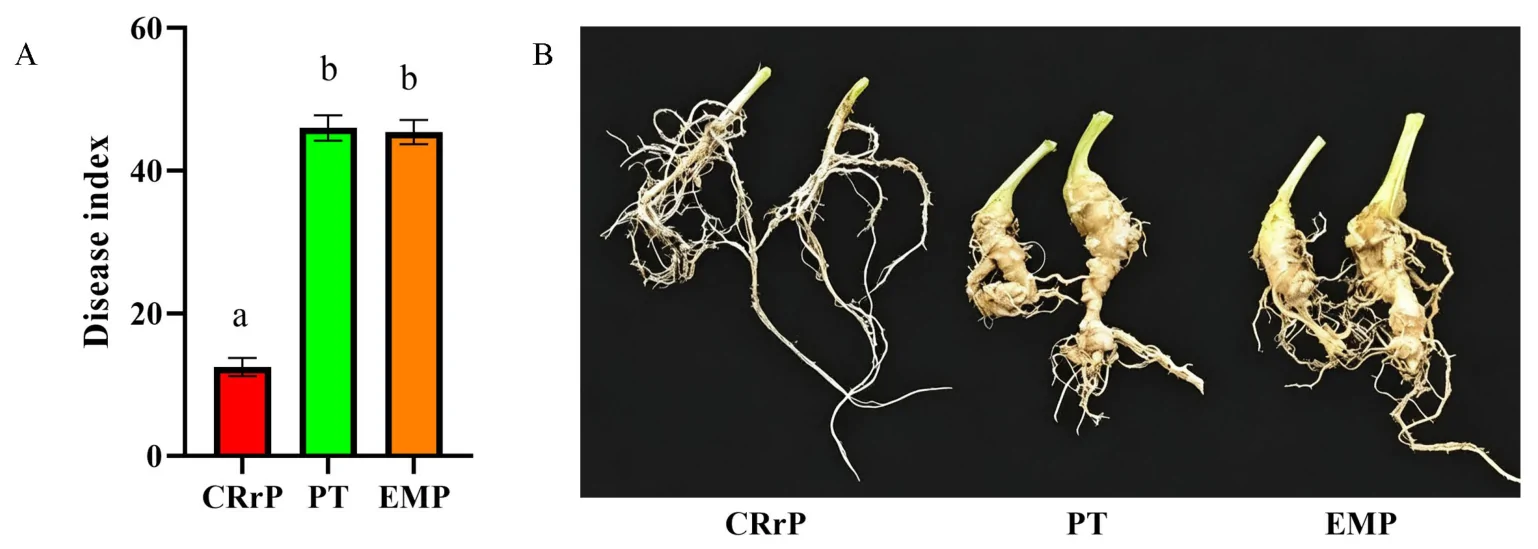
Disease-suppressive effect of the CRr protein lysate on clubroot. (**A**) Disease index across treatments; Different letters above bars indicate significant differences (*p* < 0.05). (**B**) Representative root phenotypes.

CRrP treatment significantly reduced the disease index compared to PT and EMP (empty vector and *P. brassicae* inoculation) (Figure 2A), corroborated by the absence of severe galling in CRrP roots (Figure 2B). Notably, EMP exhibited no significant protection relative to PT (*p* > 0.05), suggesting that the disease reduction is to some extent attributable to the CRr protein.

### 2.3. Alpha Diversity and Taxonomic Composition

Four treatment groups were established: CK, CRr (CRr protein-treated, non-inoculated), CRrP (CRr protein-treated followed by pathogen inoculation), and PT (pathogen-inoculated). At the class and genus levels, 66 and 503 core bacterial taxa were identified (Supplementary Figure S1), respectively, indicating that while the treatments altered community composition, they did not drastically restructure the core microbiome. In contrast, the number of unique bacterial taxa varied considerably among the treatments.

**Figure 3:**
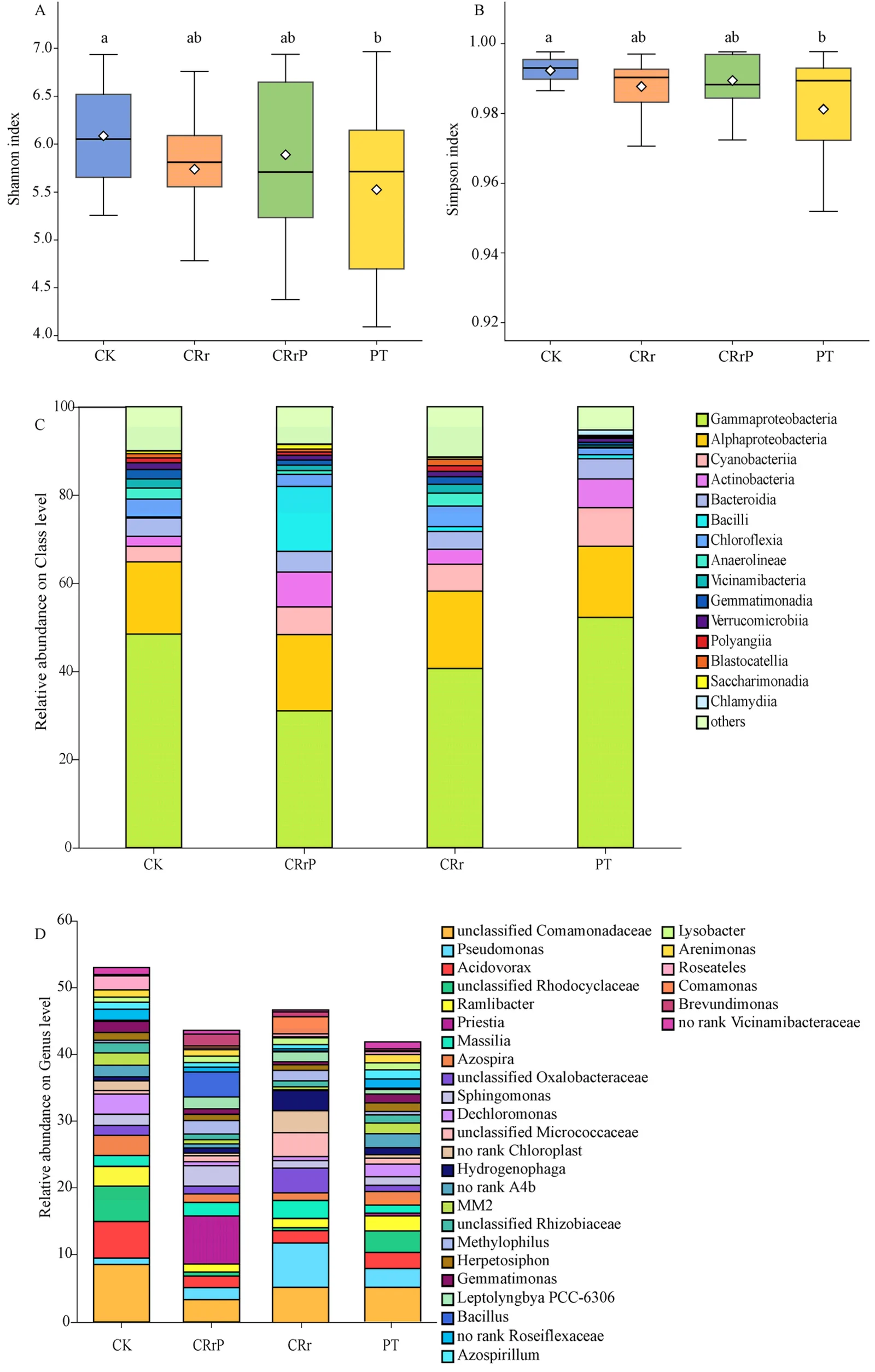
Community diversity and taxonomic structure of rhizosphere bacteria across treatments. (**A**) Shannon index. (**B**) Simpson index. (**C**) Relative abundance of the dominant bacterial classes. (**D**) Relative abundance of core genera at the genus rank. Lowercase letters denote statistically significant differences (*p* < 0.05) according to one-way ANOVA followed by Tukey’s HSD test.

The Shannon and Simpson diversity indices in the CK treatment were significantly higher than those in PT (Figure 3A,B). No significant differences were observed between Ck and CRr and CRrP, or between CRr and CRrP, all of which were comparable to each other.

At the class level, Gammaproteobacteria and Alphaproteobacteria dominated across all groups, albeit with slight variations in relative abundance. Notably, the relative abundances of Bacilli and Actinobacteria were significantly higher in the CRrP group compared to the other three treatments (Figure 3C). At the genus level, distinct taxonomic shifts were evident across treatments. While Unclassified_Comamonadaceae and *Acidovorax* predominated in CK, and *Pseudomonas* was most abundant in CRr, the CRrP treatment was characterized by the dominance of *Priestia* and *Bacillus*. In contrast, Unclassified Comamonadaceae remained the most prevalent taxon in the PT group (Figure 3D).

### 2.4. Beta Diversity and Indicator Species

**Figure 4:**
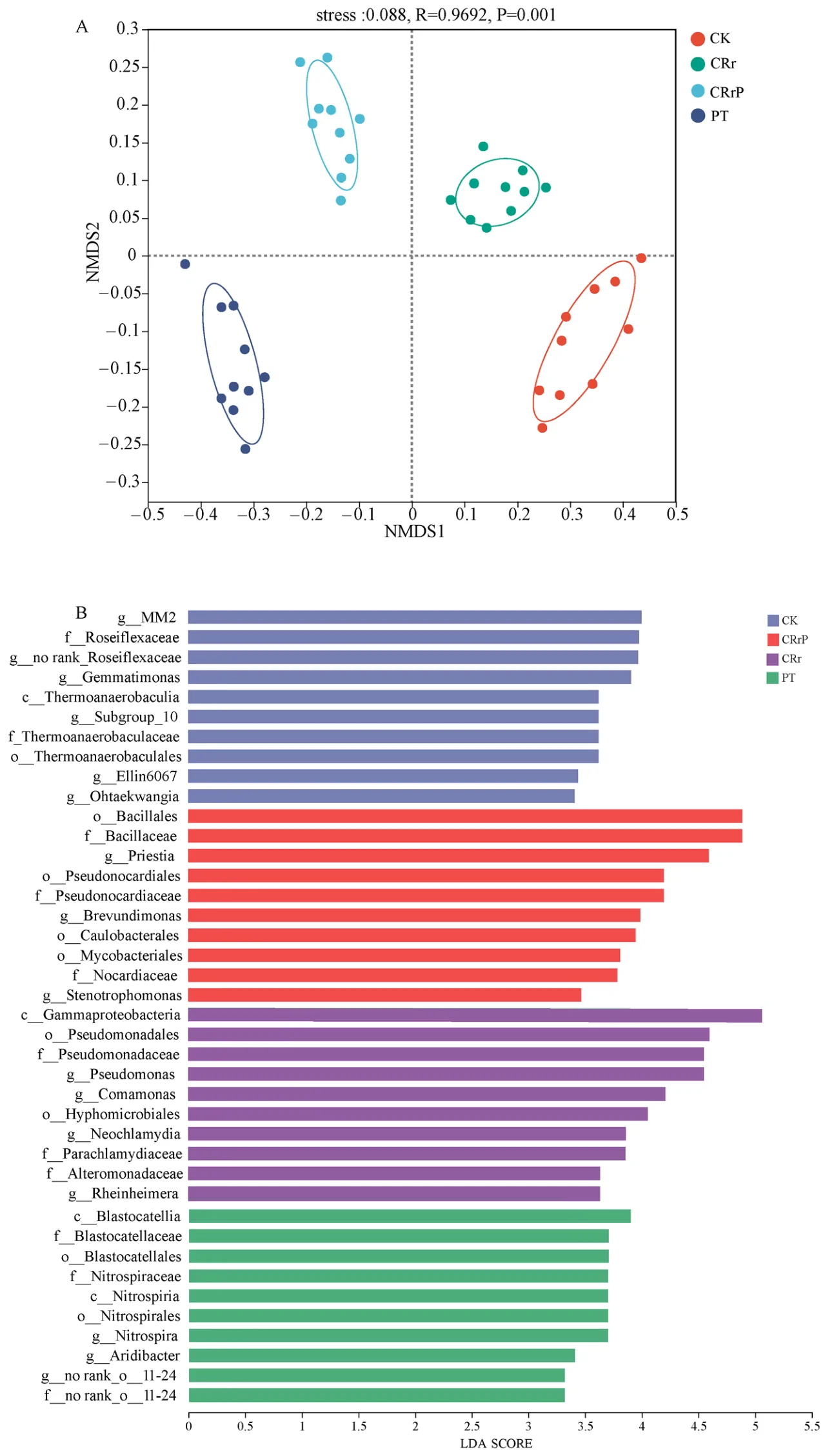
Multivariate community dissimilarity and biomarker identification. (**A**) Non-metric multidimensional scaling (NMDS) ordination based on Bray–Curtis dissimilarity; ANOSIM R = 0.9692, *p* = 0.001. (**B**) Linear Discriminant Analysis Effect Size (LEfSe) cladogram and histogram highlighting bacterial genera that differ significantly among treatments; only taxa with an LDA score exceeding 3.0 are shown.

NMDS analysis revealed significant differences in bacterial community composition among the four treatments (ANOSIM R = 0.9692, *p* = 0.001). Treatments CK and CRr clustered on the right side of the ordination plot, whereas CRrP and PT were positioned on the left (Figure 4A). Indicator species analysis identified *Gemmatimonas* and *Ohtaekwangia* as the biomarkers for Ck; *Pseudomonas*, *Comamonas*, *Neochlamydia*, and *Rheinheimera* for CRr. *Priestia*, *Brevundimonas*, and *Stenotrophomonas* for CRrP; *Nitrospira* and *Aridibacter* for PT (Figure 4B).

### 2.5. Co-Occurrence Network Analysis

**Figure 5:**
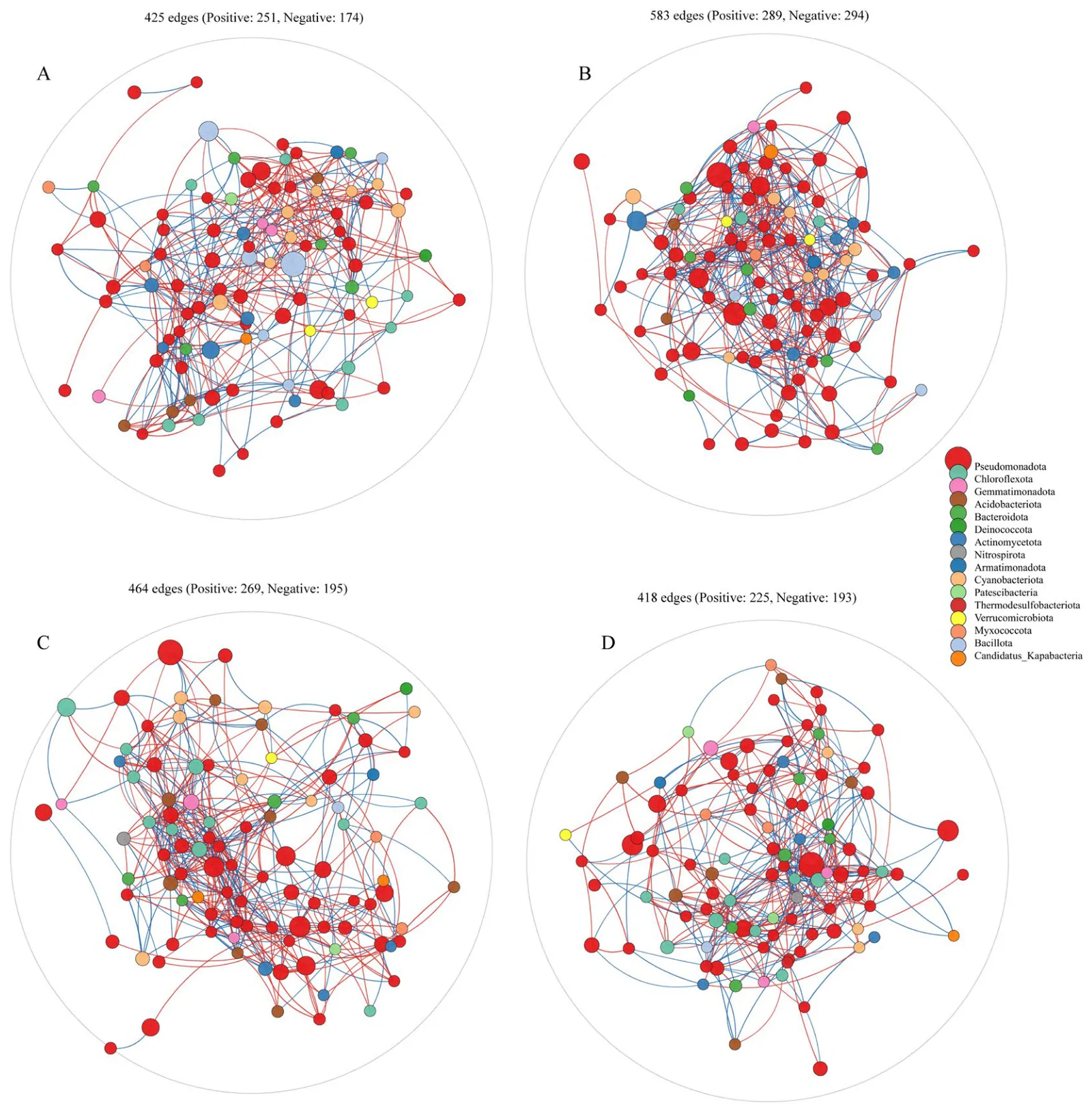
Genus-level co-occurrence networks of the rhizosphere bacterial communities. (**A**) CK group. (**B**) CRrP group. (**C**) CRr group. (**D**) PT group. Nodes represent bacterial genera, and edges represent significant correlations (*p* < 0.05) between genera. Each node represents a bacterial genus; edges denote significant correlations (*p* < 0.05) inferred by Spearman’s rank test. Node size scales with connectivity (degree). The CRr network was the most densely interconnected (583 edges), whereas CRrP (464 edges) and PT (418 edges) showed intermediate and sparse architectures, respectively, with CK being similarly sparse (425 edges). Different colors of the nodes indicate distinct taxonomic groups at the phylum level.

Co-occurrence network analysis revealed distinct topological architectures across treatments (Figure 5). Network topological complexity varied markedly across treatments. The CK group exhibited a relatively sparse network comprising 425 edges (251 positive, 174 negative) (Figure 5A). In contrast, the CRr group generated the most elaborate network topology, with 583 edges (289 positive, 294 negative) (Figure 5B). The CRrP consortium displayed intermediate complexity, with 464 edges (269 positive, 195 negative) (Figure 5C), while the PT group harbored a similarly sparse network to CK, containing 418 edges (225 positive, 193 negative) (Figure 5D).

### 2.6. Root Exudate Metabolomics

Untargeted metabolomics identified a broad spectrum of metabolites across treatments, including amino acids, organic acids, fatty acids, nucleotides, and lipids (Supplementary Figure S2A). A total of 1585 metabolites were constitutively detected across all groups (Supplementary Figure S2B). PCA effectively resolved the four treatment groups into distinct clusters with PC1 (21.40%), indicating substantial metabolic divergence (Supplementary Figure S3).

**Figure 6:**
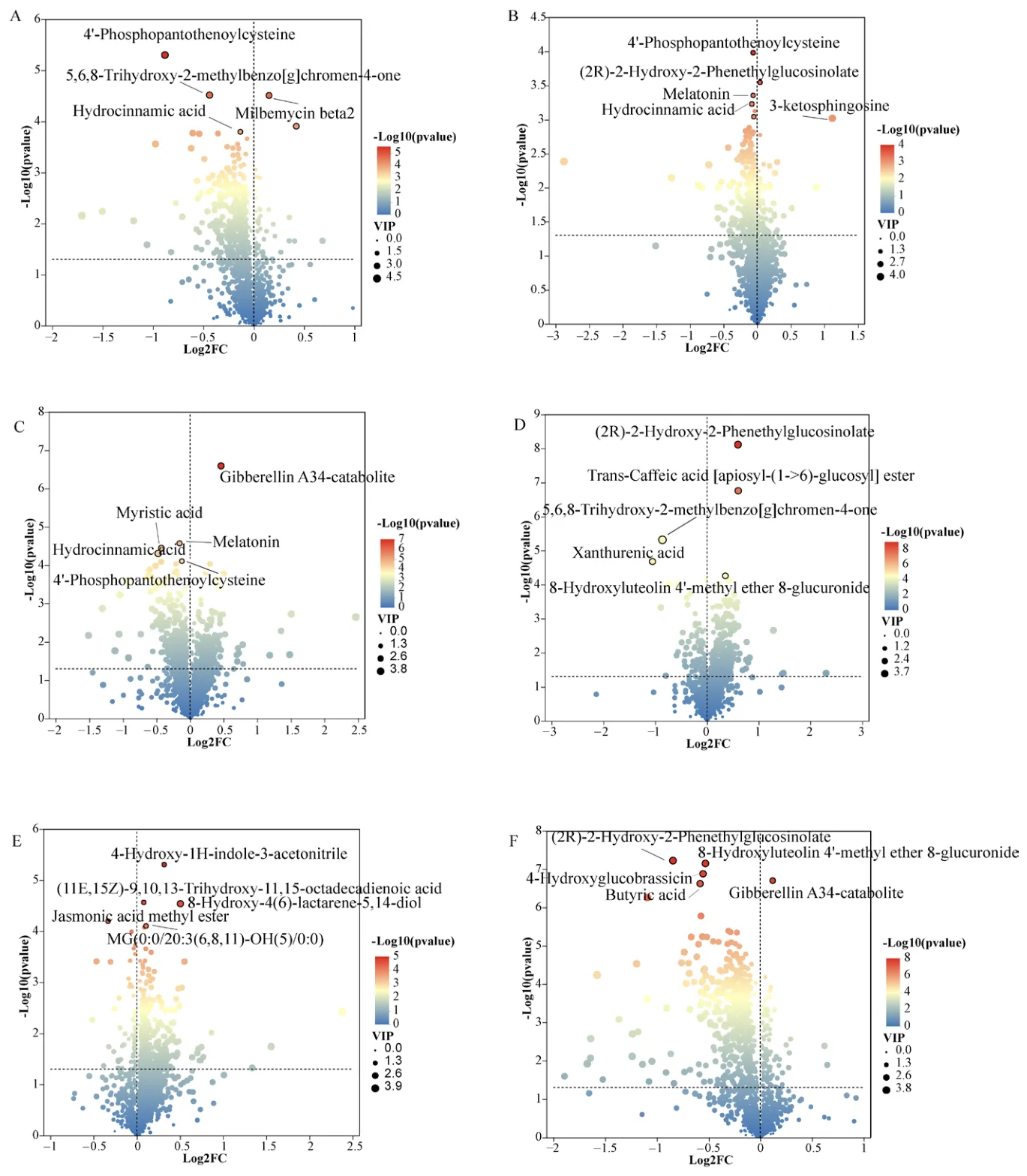
Differential metabolite profiling and metabolic reprogramming following pathogen challenge and protein application. (**A**) CK vs CRr. (**B**) CK vs CRrP. (**C**) CK vs PT. (**D**) CRaP vs CRr. (**E**) PT vs CRr. (**F**) CRrP vs PT. Volcano plots show the distribution of significantly altered metabolites; red and blue points indicate up- and down-accumulated compounds, respectively. Differentially abundant metabolites were selected using a VIP threshold ≥ 1 in the OPLS-DA model together with a fold change ≥ 2 or ≤ 0.5.

Differential metabolite analysis (VIP > 1.5, *p*< 0.05) revealed treatment-specific patterns of metabolic reprogramming. Compared to CK, CRr exhibited elevated levels of 5,6,8-trihydroxy-2-methylbenzo[g]chromen-4-one and milbemycin beta2, while Ck retained higher 4′-phosphopantothenoylcysteine and hydrocinnamic acid (Figure 6A). Relative to CK, CRrP uniquely accumulated (2R)-2-hydroxy-2-phenethylglucosinolate, whereas CK showed higher melatonin (Figure 6B). Compared to CK, PT was marked by enriched gibberellin A34-catabolite (Figure 6C). Versus CRr, CRrP additionally accumulated trans-caffeic acid [apiosyl-(1→6)-glucosyl] ester alongside the aforementioned glucosinolate (Figure 6D). Contrasting CRr with PT revealed PT-specific enrichment of 4-hydroxy-1H-indole-3-acetonitrile, the (11E,15Z)-9,10,13-trihydroxy-11,15-octadecadienoic acid, and 8-hydroxy-4(6)-lactarene-5,14-diol, while CRr maintained higher jasmonic acid methyl ester (Figure 6E). Finally, compared to PT, CRrP presented elevated (2R)-2-hydroxy-2-phenethylglucosinolate, 8-hydroxyluteolin 4′-methyl ether 8-glucuronide, and 4-hydroxyglucobrassicin, whereas PT retained higher Gibberellin A34-catabolite (Figure 6F).

### 2.7. Integrated Microbiome-Metabolome Analysis

**Figure 7:**
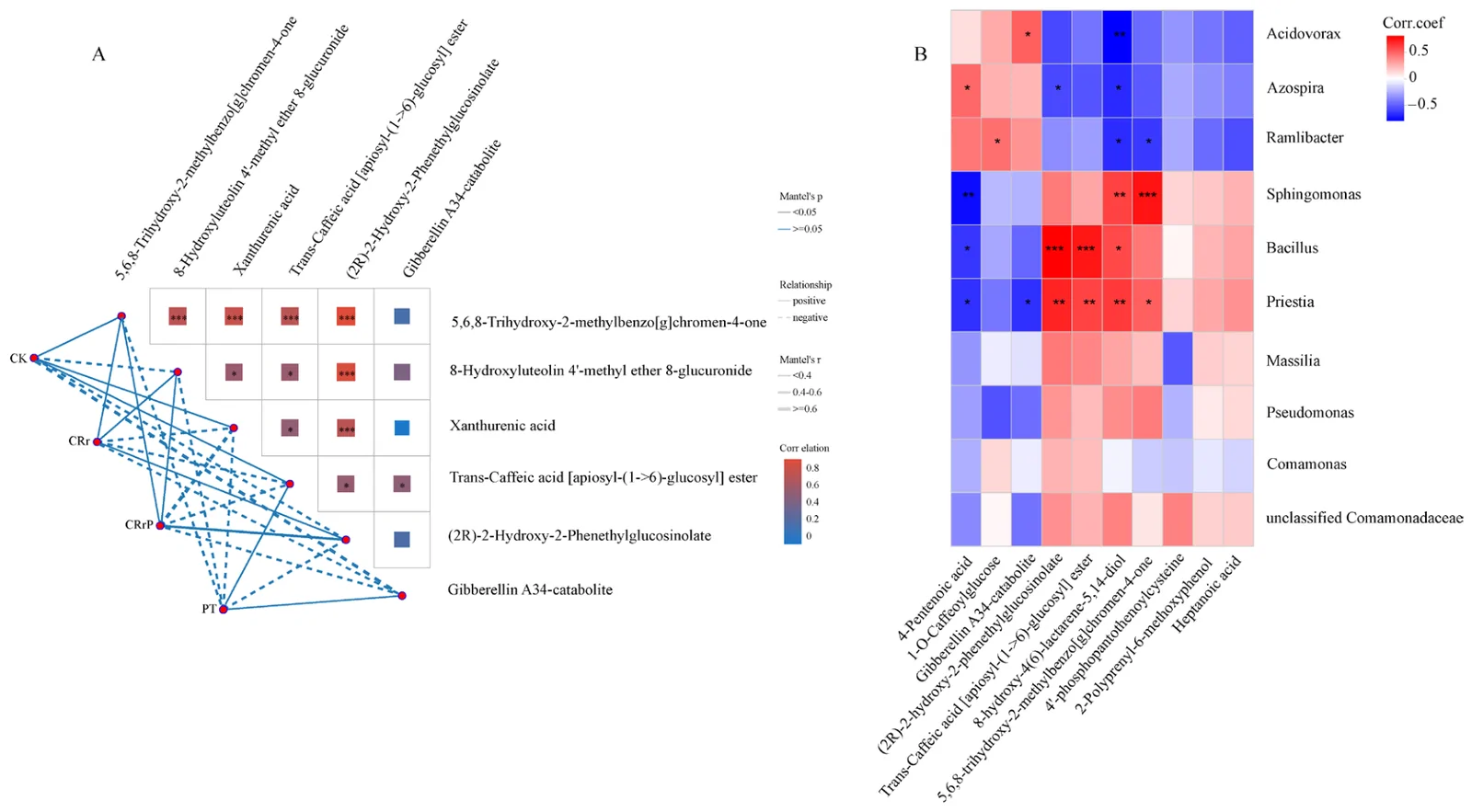
Integrated microbiome–metabolome association analysis. (**A**) Mantel test evaluating the correlation between rhizosphere bacterial community structure (Bray–Curtis dissimilarity) and the abundance profiles of differentially accumulated root-exudate metabolites. (**B**) Heat map depicting Spearman rank correlations between differentially abundant bacterial genera and key host-defense-associated metabolites; red and blue cells denote positive and negative associations, respectively. Asterisks indicate significant differences at *p* < 0.05 (*), *p* < 0.01 (**), and *p* < 0.001 (***), respectively.

Procrustes analysis revealed a modest correlation between rhizosphere microbiome composition and root exudate profiles (M^2^ = 0.788, *p* = 0.025; Supplementary Figure S4), confirming strong microbe-metabolite linkages. Mantel tests further demonstrated that the CRr and CRrP groups exhibited positive correlations with the majority of differential metabolites, including (2R)-2-hydroxy-2-phenethylglucosinolate, Trans-Caffeic acid [apiosyl-(1->6)-glucosyl] ester, 8-Hydroxyluteolin 4′-methyl ether 8-glucuronide, and 5,6,8-Trihydroxy-2-methylbenzo[g]chromen-4-one (Figure 7A). Conversely, PT showed positive associations with the gibberellin A34 catabolite.

Spearman’s correlation analysis delineated distinct genus-level interaction networks (Figure 7B). Specifically, *Bacillus* and *Priestia* exhibited strong positive associations with key host defense metabolites, including (2R)-2-hydroxy-2-phenethylglucosinolate, trans-caffeic acid [apiosyl-(1→6)-glucosyl] ester, and 8-hydroxy-4(6)-lactarene-5,14-diol. In contrast, Sphingomonas correlated significantly with 5,6,8-trihydroxy-2-methylbenzo[g]chromen-4-one.

## 3. Discussion

### 3.1. Applied CRr Protein Moves Systemically Through the Root

The past decade has witnessed a paradigm shift in plant disease management—from gene-introgressive resistance breeding toward protein-direct immunity [10,11]. Our study extends this concept to *P. brassicae*, a soil-borne protist for which traditional CR introgression is hindered by linkage drag, multigenicity, and the rapid emergence of virulence shifts [8]. By fusing the *Crr1a* TIR-NBS-LRR coding sequence—originally cloned from *B. rapa* A8—to an RXLR translocation motif derived from *P. sojae* Avr1b [12], we achieved systemic root movement within 24 h, with fluorescence signals progressing from cortical foci (1–3 hpi) to the stele (12 hpi) and full-tissue saturation (24 hpi). This spatiotemporal trajectory mirrors RXLR-mediated effector entry into host cells [12]. Crucially, this approach achieved substantive clubroot suppression (73.6%) in *B. juncea* var. tumida. This spatiotemporal trajectory is consistent with models of RXLR-mediated delivery [12]. Crucially, the crude lysate treatment was associated with substantial clubroot suppression in *B. juncea* var. tumida.

To address whether this protection was mediated by the CRr protein or reflected a nonspecific response to bacterial components, we included an equal total protein amount of *E. coli* lysate derived from the pET-28a(+) backbone—which was evaluated for clubroot disease phenotype. The EMP treatment showed no significant difference in disease index compared to the pathogen-only (PT) control (*p* > 0.05), confirming that the observed disease reduction is attributable to the CRr protein rather than to general bacterial PAMPs, endotoxins, or the vector backbone itself. This result strengthens the conclusion that RXLR-mediated delivery of an exogenous NLR resistance protein can confer targeted protection without genetic modification of the crop.

Nevertheless, several important caveats remain. First, the current study used crude bacterial lysate without protein purification; future work should incorporate purified CRr protein to eliminate any residual contribution from trace bacterial components. Second, additional controls—including heat-inactivated lysate and an RXLR–eGFP-only fusion protein—will further consolidate the evidence for specific CRr protein activity. Third, true cytoplasmic entry of the intact fusion protein and its interaction with endogenous immune nodes remain to be formally validated using confocal microscopy with plasmolysis, anti-GFP immunoblots of cytosolic fractions, and cell-autonomous immunity assays.

It should also be noted that the EMP was incorporated as an additional control and was therefore assessed exclusively for disease phenotype specificity; microbiome profiling and metabolomic analyses remained restricted to the original four core treatments (CK, CRr, CRrP, PT). Extending the multi-omics pipeline to include the EMP group in future studies will clarify whether any subtle microbial or metabolic shifts are uniquely driven by the CRr protein versus general bacterial elicitors.

### 3.2. Exudate Metabolite Profiles Are Consistent with a Defense-Primed State

Our core finding is that CRr protein pretreatment is associated with metabolic changes that are consistent with activation of defense pathways. While the salicylic acid–driven branch promotes indole glucosinolate accumulation and gibberellin catabolism [18], the jasmonic acid–driven branch simultaneously enhances the production of aromatic glucosinolates, oxylipins, and methyl jasmonate [19]. Notably, CRrP-treated plants exhibited a marked accumulation of specific defense-related metabolites, namely (2R)-2-hydroxy-2-phenethylglucosinolate and 4-hydroxyglucobrassicin. This selective enrichment of distinct glucosinolate species suggests a targeted reprogramming of secondary metabolism associated with CRr-mediated resistance. Consistent with this notion, indolic and aromatic glucosinolates have been implicated in Brassica defense against *P. brassicae* [20], and resistant *B. rapa* genotypes often show altered glucosinolate profiles [21]. Whether the CRr lysate recapitulates the metabolite state of CRa/CRb lines remains to be confirmed [22]. The observed shifts in root exudate-associated metabolites—including jasmonic acid methyl ester and glucosinolate derivatives—suggest that CRr treatment may prime defense-related metabolic pathways. However, we acknowledge that these exudate profiles represent secreted/metabolite-associated signatures rather than direct measurements of intracellular hormone concentrations within gall tissues. Future studies should include qRT-PCR validation of defense-related genes to quantitatively assess the immune priming effect of CRr treatment.

Another clear sign of this defense shift is the trade-off between the growth hormone gibberellin and the defense hormone salicylic acid. Plants infected only with the pathogen (the PT group) retained high levels of a gibberellin breakdown product called gibberellin A34 catabolite. In contrast, CRrP-treated plants suppressed this gibberellin buildup while ramping up production of glucosinolates and flavonoids. We know that *P. brassicae* takes advantage of host gibberellin and auxin to trigger clubroot gall formation [23]. Our results show that CRr protein frees the SA/JA defense systems from the suppressive effects of gibberellin and auxin. CRr treatment also raised levels of two key phenylpropanoid/flavonoid compounds: trans-caffeic acid [apiosyl-(1→6)-glucosyl] ester and 8-hydroxyluteolin 4′-methyl ether 8-glucuronide. While these metabolites are detected in root exudate collections, we note that the exudate preparation method cannot exclude contributions from intracellular metabolites released during the 12 h exudation period. Therefore, these profiles should be interpreted as exudate-associated metabolic signatures rather than definitive measurements of intracellular hormone levels within root tissues or galls. Whether these compounds directly suppress *P. brassicae* or serve as signals recruiting beneficial rhizobacteria requires further investigation.

### 3.3. Microbiome Remodeling: From Host Exudates to Candidate Beneficial Groups

Root exudates are primary determinants of rhizosphere microbial assembly, acting as chemical signals that shape the composition and function of the plant-associated microbiome. Indicator taxa for CRrP included *Priestia*, *Brevundimonas*, and *Stenotrophomonas*, whereas PT was characterized by *Nitrospira* and *Aridibacter*. Notably, the enrichment of Priestia (a recently reclassified clade within the former *B. megaterium* group) and Bacillus (the dominant genus in CRr) in protein-treated groups aligns with the substantial reduction in disease index. These genera are well-documented for their plant growth-promoting and biocontrol capabilities, and their recruitment likely contributed directly to the observed clubroot suppression [24,25].

Co-occurrence network analysis provided additional insights into the structural complexity of these communities. The CRr treatment generated the most elaborate network topology (583 edges), followed by CRrP (464 edges), while PT and CK harbored sparser networks (418 and 425 edges, respectively). The high connectivity in CRr and CRrP networks, particularly the increased proportion of positive interactions, suggests that CRr protein application enhanced microbial synergies and ecological cohesion. Such tightly connected networks are generally considered more resilient to environmental perturbations and pathogen invasion [26,27]. Conversely, the simplified network in PT mirrors the typical dysbiosis associated with disease states, where keystone taxa are lost and network stability declines.

The integration of microbiome and metabolome data unequivocally linked these ecological changes to host metabolic outputs. Procrustes analysis revealed significant congruence between bacterial community composition and root exudate profiles (M^2^ = 0.788, *p* = 0.025), confirming a strong microbe–metabolite crosstalk. Spearman correlation analysis further delineated specific bipartite interactions, showing that *Bacillus* and *Priestia* exhibited strong positive associations with key defense metabolites, including (2R)-2-hydroxy-2-phenethylglucosinolate, trans-caffeic acid [apiosyl-(1→6)-glucosyl] ester. These metabolites, uniquely or significantly upregulated by CRr protein, likely serve as chemoattractants or growth substrates that recruit beneficial bacteria to the rhizosphere. For instance, aromatic glucosinolates and phenylpropanoid derivatives have been shown to modulate microbial recruitment in Brassica species [28], and their enrichment in CRrP provides a plausible mechanistic link between host metabolic shifts and microbiome remodeling.

It should be noted that pathogen inoculation—not protein treatment—was the primary driver of overall community structure (evidenced by the large NMDS1 shift between CK and PT). Protected plants (CRrP) maintained a pathogen-altered overall community structure while selectively enriching specific taxa, rather than reverting to the healthy CK baseline state.

## 4. Materials and Methods

### 4.1. Plant Material and Germination

The non-resistant tumorous stem mustard cultivar ‘Yong’an Xiaoye’, obtained from the Fuling Academy of Agricultural Sciences (Chongqing, China), was used as the plant material. Surface-sterilized seeds were germinated on moist filter paper within sterile Petri dishes. Following three rinses with sterile water after a 5 min immersion in 75% ethanol, the seeds were incubated at 23 °C under a 16 h/8 h light/dark cycle. Healthy, aseptic seedlings with fully expanded cotyledons were selected after approximately 5 days for downstream applications, with each dish receiving 40 mL of half-strength Hoagland solution.

### 4.2. Heterologous Expression and Purification of Recombinant Protein

To assess the intracellular delivery efficacy of the RXLR motif, we engineered a synthetic construct derived from the *Phytophthora sojae* Avr1b effector [12]. This design integrates a 42 bp RXLR-EER translocation domain, a C-terminally truncated eGFP reporter, and the full-length *Brassica rapa* Crr1a (3749 bp; GenBank accession AB605024.1), culminating in a 160 kDa fusion protein. Following commercial synthesis (BGI, Shenzhen), the cassette was ligated into pET-28a (+), generating pET28a-eGFP-CRr. After heat-shock transformation into *E. coli* BL21(DE3), integrants were authenticated via colony PCR and Sanger sequencing.

Overnight starter cultures (37 °C, 200 rpm) were initiated in kanamycin-supplemented LB medium. Working cultures, inoculated at a 1:100 dilution, were grown to an OD_600_ of 0.6–0.8, at which point a glycerol stock (50% *v*/*v*) was archived at -80 °C. Concurrently, protein expression was induced with 1 mM IPTG at 16 °C overnight. Post-induction, cells were pelleted, lysed by sonication in PBS, and clarified by centrifugation (12,000× *g*, 20 min, 4 °C). The soluble fraction was electrophoresed on a 10% SDS-PAGE gel to validate the fusion product and retained on ice for subsequent applications.

As a negative control, *E. coli* BL21(DE3) carrying the empty vector pET-28a(+) was cultured, induced, and lysed under identical conditions. Total protein concentration of the empty vector lysate was determined by Bradford assay and adjusted to an equal amount (approximately 2.0 mg/mL total protein) relative to the CRr protein crude lysate. This empty vector control was prepared specifically to exclude nonspecific effects of bacterial components or the vector backbone on clubroot disease phenotype; it was not included in the microbiome or metabolome profiling pipelines, which were conducted on the original four core treatments prior to empty vector incorporation.

### 4.3. Assessment of Root Protein Movement Through Root

Seedlings were transferred into 2 mL microcentrifuge tubes containing 1.0 mL of the crude protein extract, ensuring complete submersion of the root systems. Stems were secured through perforated caps, and plants were maintained at 23 °C under a 16 h photoperiod. Roots were harvested at 1, 3, 6, 9, 12, and 24 h post-immersion. Following five rigorous rinses (30 s each) with sterile water, root tips and hair zones were excised, mounted on slides, and examined immediately under epifluorescence microscopy to visualize GFP signals and assess protein adherence and infiltration [29].

### 4.4. Propagation of P. brassicae Resting Spores

Resting spores were isolated from galls of naturally infected stem mustard plants. After thorough washing, diseased tissues were surface-sterilized in 75% ethanol for 10 min, followed by five sterile water rinses. Sterile gall tissues were homogenized in water, and the slurry was filtered through eight layers of cheesecloth. The filtrate was subjected to sequential centrifugation cycles (5000× *g*, 10 min, 4 °C), with spore pellets being washed three times to ensure purity. The final spore concentration was adjusted to 1.0 × 10^8^ spores/mL using a hemocytometer and stored at 4 °C pending inoculation [30]. Spore concentrations were quantified using a hemocytometer at 400× magnification under a light microscope, with three technical replicates per sample. The virulence of *P. brassicae* was assessed using the Sinitic clubroot differential system [31].

### 4.5. Bioassay Experimental Design

Five treatment regimes were established: (i) CRrP: 24 h CRr protein lysate priming followed by transplantation and *P. brassicae* inoculation; (ii) CRr: 24 h protein priming followed by transplantation and mock inoculation (sterile nutrient solution); (iii) PT: 24 h immersion in sterile PBS followed by transplantation and pathogen inoculation; (iv) CK: 24 h PBS immersion followed by transplantation and mock inoculation; and (v) EMP (empty vector and *P. brassicae* inoculation): 24 h treatment with an equal total protein amount of *E. coli* lysate derived from the empty vector pET-28a(+), prepared and quantified as described above, followed by transplantation and *P. brassicae* inoculation. Each treatment comprised 10 biological replicates (pots, 3 plants/pot), thus yielding a total of 50 pots (150 plants) across the experiment. Five weeks post-inoculation, whole plants were harvested. Disease incidence and indices were scored based on a standardized grading scale [30]. Disease severity was graded on a 0–4 scale: 0 = no galls; 1 = 1–25% of root system galled; 2 = 26–50% galled; 3 = 51–75% galled; 4 = 76–100% galled. The disease index (DI) was calculated as: DI = [Σ (number of plants in each grade × grade value)] / (total number of plants × 4) × 100. Rhizosphere soil (adhering soil) was collected for microbial analysis, while root exudates were obtained by rinsing the harvested roots.

### 4.6. Rhizobacterial Community Profiling via Illumina miSeq

Rhizosphere soil samples were collected from three pooled plants to constitute one biological replicate, with 10 replicates per treatment. Total metagenomic DNA was extracted from 0.5 g of rhizosphere soil using the FastDNA™ SPIN Kit (MP Biomedicals, Irvine, CA, USA). DNA integrity and concentration were assessed via agarose gel electrophoresis and a NanoDrop 2000 spectrophotometer (Thermo Fisher Scientific, Wilmington, DE, USA), respectively. The V3–V4 hypervariable region of the bacterial 16S rRNA gene was amplified using primers 338F (5′-ACTCCTACGGGAGGCAGCAG-3′) and 806R (5′-GGACTACHVGGGTWTCTAAT-3′). Purified amplicons were pooled at equimolar ratios to construct libraries following Illumina’s standard 16S rRNA gene sequencing protocol. Sequencing was performed on an Illumina MiSeq PE300 platform at Majorbio Bio-Pharm Technology Co., Ltd. (Shanghai, China).

Raw reads were demultiplexed and quality-filtered using q2-cutadapt and q2-dada2 in QIIME2 [32]. Amplicon Sequence Variants (ASVs) were inferred. Taxonomy was assigned using the SILVA 138 database. Chloroplast and mitochondrial sequences were filtered out prior to diversity analyses. Alpha diversity was assessed using the Shannon and Simpson indices, and statistical differences among treatments were evaluated by the Kruskal–Wallis test followed by Dunn’s post hoc test (*p* < 0.05). Venn diagrams were constructed to illustrate the overlap of bacterial taxa at the class and genus levels. Relative abundances at these taxonomic levels were subsequently calculated. Beta diversity was visualized using Non-metric Multidimensional Scaling (NMDS) based on Bray–Curtis dissimilarity. Finally, differentially abundant genera were identified via Linear Discriminant Analysis Effect Size (LEfSe), employing an LDA score threshold > 3.0 and a Kruskal–Wallis test (*p* < 0.05) [33]. To visualize the network structure for the rhizosphere bacteria, a network based on the top 100 OTUs was drawn using the Networkx software on the Majorbio I-Sanger Cloud Platform [34].

### 4.7. Non-Targeted Metabolomic Profiling of Root Exudates

Root exudates were collected by immersing intact [35], root systems in 100 mL of Milli-Q water for 12 h under light (25 ± 0.5 °C). For each treatment, five independent biological replicates were established, with each replicate comprising pooled root exudates derived from six individual plants. The exudate fluid was filtered (0.22 µm), flash-frozen in liquid nitrogen, and lyophilized. Dried samples were reconstituted in 70% methanol containing internal standards prior to LC-MS analysis. Quality Control (QC) samples were interspersed throughout the run sequence. Metabolites were identified via the MetWare database and quantified in Multiple Reaction Monitoring (MRM) mode [36]. Multivariate statistical analyses, including PLS-DA and 200-cycle permutation testing, were conducted to validate model robustness [37]. Differential metabolites were screened based on VIP ≥ 1.5 and |fold change| ≥ 2 and visualized via volcano plots.

### 4.8. Integrative Correlation Analyses

To delineate the interplay between the rhizosphere microbiome and host metabolism, we first assessed the overall congruence between bacterial community structure and root-exudate metabolite profiles using Procrustes analysis. Because the 16S amplicon (10 pooled replicates; 3 plants per pool) and metabolomics (5 pooled replicates; 6 plants per pool) datasets differed in sample pooling and replicate numbers, Procrustes fitting was performed on treatment-level mean profiles rather than on matched individual samples; accordingly, these results are intended as qualitative, exploratory corroboration and should be interpreted with caution. The global association between the two datasets was then evaluated with a Mantel test (Spearman-based distance correlation). Finally, Spearman correlation analysis was used to resolve specific bipartite interactions between bacterial genera and individual metabolites. Significance was assigned using a joint threshold of |ρ| > 0.6 and a two-tailed *p* < 0.05 [38].

## 5. Conclusions

Exogenous application of crude CRr protein lysate suppresses clubroot in tumorous stem mustard through systemic protein dissemination, host metabolic shifts, and rhizosphere microbiome restructuring toward beneficial taxa including *Bacillus* and *Priestia*. The inclusion of an EMP confirmed that this protection is associated with the RXLR–CRr fusion rather than with nonspecific bacterial components (*p* > 0.05 vs. pathogen-only). These findings establish RXLR-mediated exogenous NLR delivery as a viable non-transgenic strategy for soil-borne disease management and provide a foundation for microbiome-oriented green control approaches. Future work with purified protein, heat-inactivated controls, and RXLR–eGFP fusions will further consolidate the mechanistic basis of this technology.

## Data Availability

All data supporting this study are included in the manuscript and Supplementary Files. Additional inquiries should be directed to the corresponding author. The raw 16S rRNA reads have been uploaded to the NCBI Sequence Read Archive (SRA) database with the accession number PRJNA1495499.

## Supplementary Materials

The following supporting information can be downloaded at: https://www.mdpi.com/article/10.3390/plants15213373/s1, Figure S1: Core bacterial taxa identified across all treatment groups at the class (A) and genus (B) levels. Core taxa were defined as those present in all biological replicates within a treatment at ≥ 0.01% relative abundance; Figure S2: Compositional landscape of root exudate metabolomes. (A) Chemical family distribution of detected metabolites, categorized by structural taxonomy. (B) Inter-group metabolite sharing patterns among the four treatments, displayed as a Venn diagram. Figure S3: Principal component analysis of root exudate metabolic profiles. Samples from each treatment form distinct clusters, indicating treatment-specific metabolic reprogramming. Figure S4: Procrustes superimposition of rhizosphere bacterial community structure (based on PCA of differential genera) and root exudate metabolic profiles. The analysis reveals a significant overall correlation between microbial assemblages and host metabolic outputs across treatments.

## Abbreviations

The following abbreviations are used in this manuscript:

RXLR	Arg-X-Leu-Arg
EMP	Empty vector and *P. brassicae* inoculation
ASVs	Amplicon Sequence Variants
NMDS	Non-metric Multidimensional Scaling
LEfSe	Linear Discriminant Analysis Effect Size
MRM	Multiple Reaction Monitoring
