# Supplementary material for: RXLR-Mediated Delivery of Exogenous CRr Protein Reduces Clubroot in Tumorous Stem Mustard and Reshapes the Rhizosphere Microbiome
Source: Plants (Basel). 2026 Nov 1;15(21):3373. doi: 10.3390/plants15213373 (PMC13766053; doi:10.3390/plants15213373)
Supplement: Supplementary file 1 [file plants-15-03373-s001.zip › Figure S1.pdf]

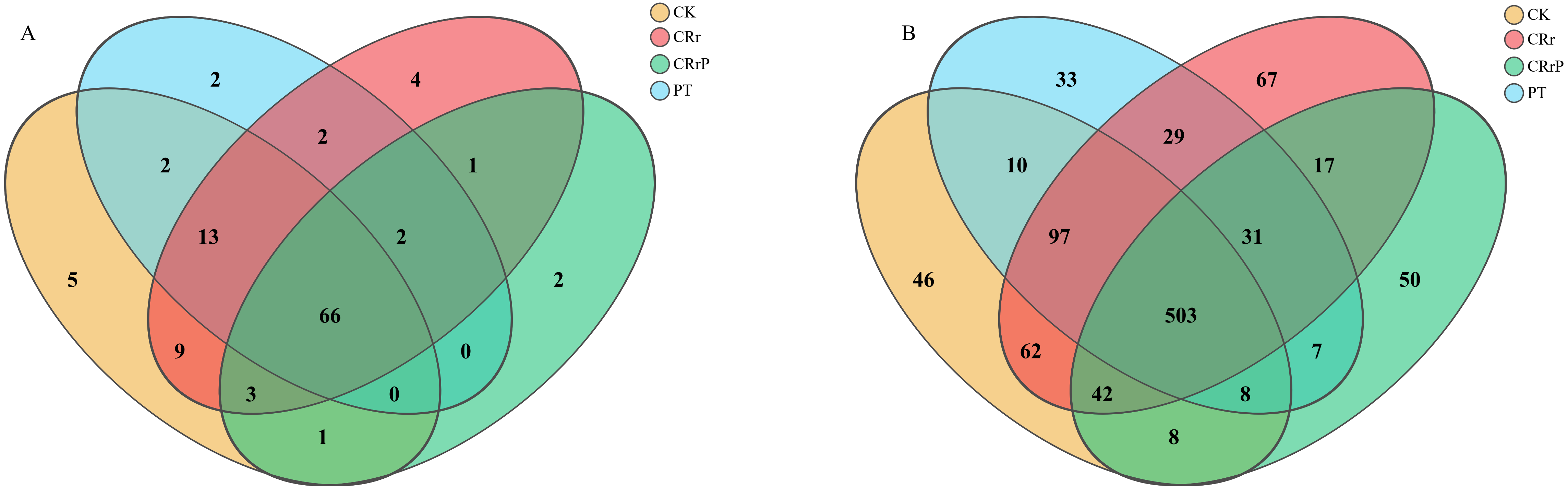

A
CK
CRr
CRrP
PT
2
4
2
2
1
13
2
5
2
66
9
0
3
0
1
B
CK
CRr
CRrP
PT
33
67
29
10
17
97
31
46
50
503
62
7
42
8
8
