# Supplementary material for: RXLR-Mediated Delivery of Exogenous CRr Protein Reduces Clubroot in Tumorous Stem Mustard and Reshapes the Rhizosphere Microbiome
Source: Plants (Basel). 2026 Nov 1;15(21):3373. doi: 10.3390/plants15213373 (PMC13766053; doi:10.3390/plants15213373)
Supplement: Supplementary file 1 [file plants-15-03373-s001.zip › Figure S2.pdf]

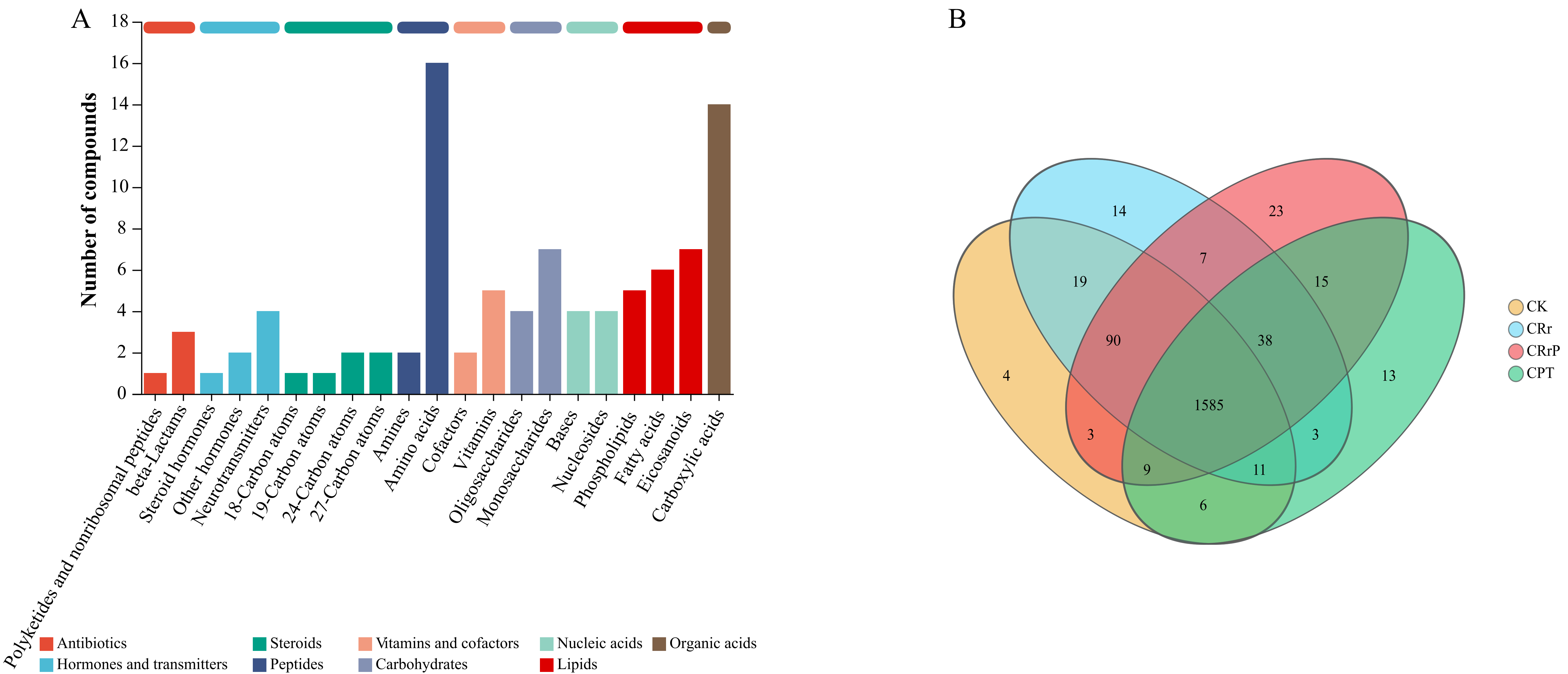
A
B
Number of compounds
0
2
4
6
8
10
12
14
16
18
Polyketides and nonribosomal peptides
beta-Lactams
Steroid hormones
Other hormones
Neurotransmitters
18-Carbon atoms
19-Carbon atoms
24-Carbon atoms
27-Carbon atoms
Amines
Amino acids
Cofactors
Vitamins
Oligosaccharides
Monosaccharides
Bases
Nucleosides
Phospholipids
Fatty acids
Eicosanoids
Carboxylic acids
Antibiotics
Hormones and transmitters
Steroids
Peptides
Vitamins and cofactors
Carbohydrates
Nucleic acids
Lipids
Organic acids
14
23
7
19
15
90
38
4
13
1585
3
3
9
11
6
CK
CRr
CRrP
CPT
