# Supplementary material for: RXLR-Mediated Delivery of Exogenous CRr Protein Reduces Clubroot in Tumorous Stem Mustard and Reshapes the Rhizosphere Microbiome
Source: Plants (Basel). 2026 Nov 1;15(21):3373. doi: 10.3390/plants15213373 (PMC13766053; doi:10.3390/plants15213373)
Supplement: Supplementary file 1 [file plants-15-03373-s001.zip › Figure S3.pdf]

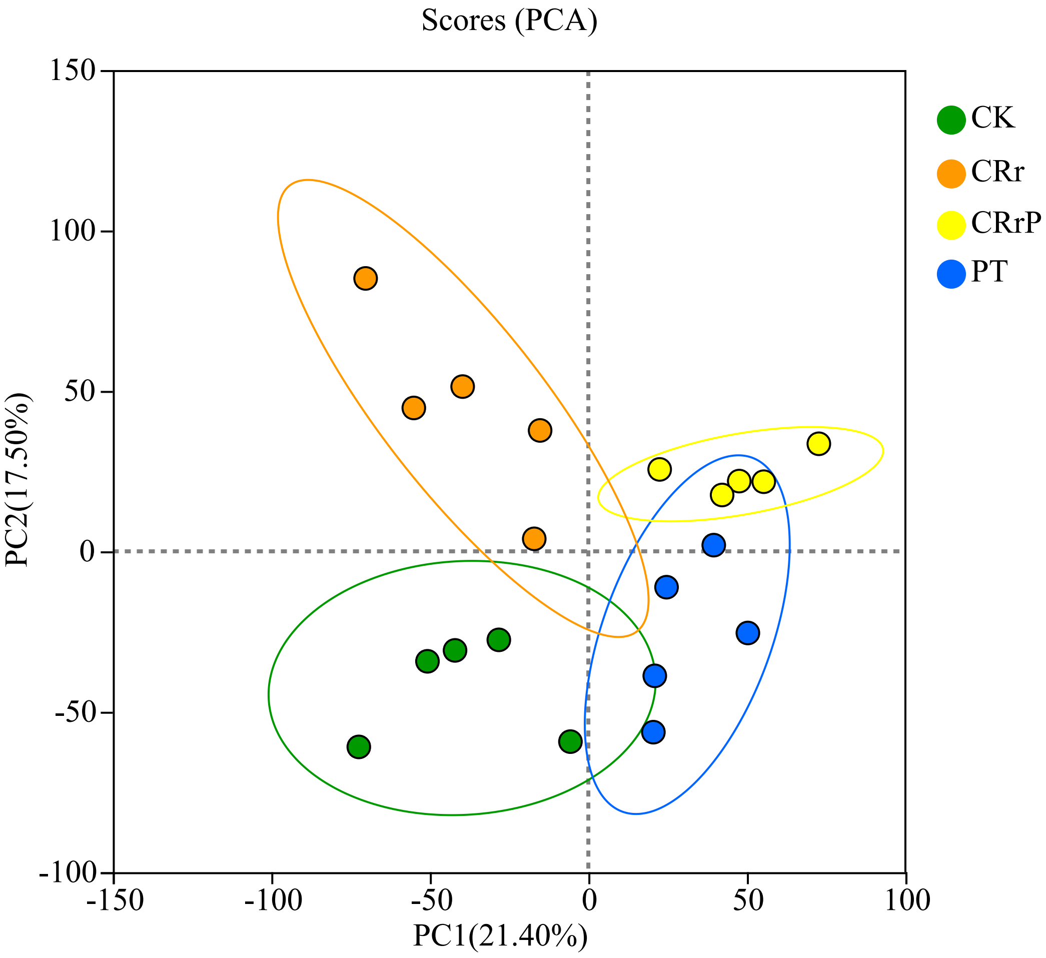

Scores (PCA)
150
100
50
0
-50
-100
-150
-100
-50
0
50
100
PC1(21.40%)
PC2(17.50%)
CK
CRr
CRrP
PT
