# Supplementary material for: RXLR-Mediated Delivery of Exogenous CRr Protein Reduces Clubroot in Tumorous Stem Mustard and Reshapes the Rhizosphere Microbiome
Source: Plants (Basel). 2026 Nov 1;15(21):3373. doi: 10.3390/plants15213373 (PMC13766053; doi:10.3390/plants15213373)
Supplement: Supplementary file 1 [file plants-15-03373-s001.zip › Figure S4.pdf]

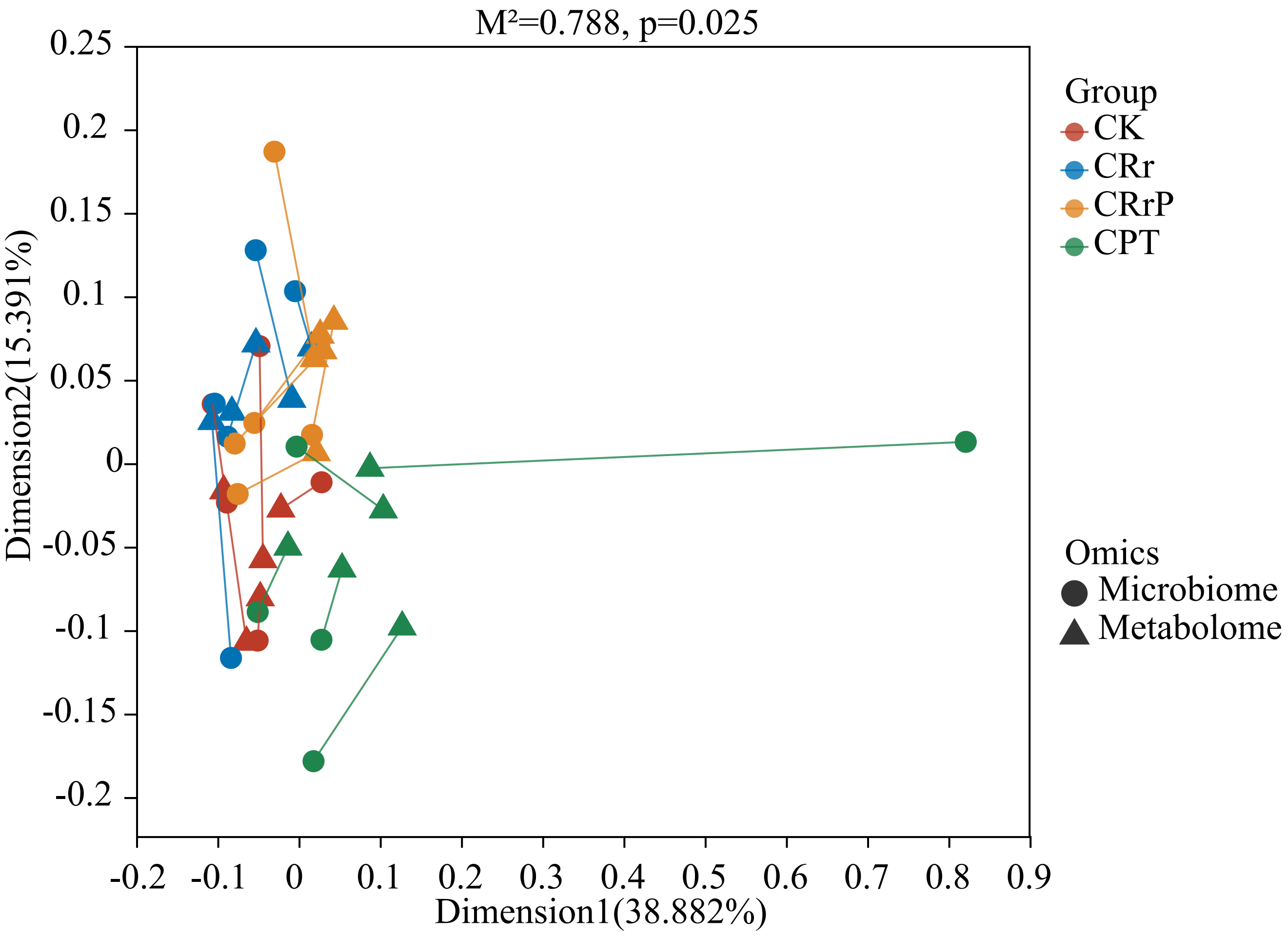

M²=0.788, p=0.025
0.25
0.2
0.15
0.1
0.05
0
-0.05
-0.1
-0.15
-0.2
Dimension2(15.391%)
-0.2
-0.1
0
0.1
0.2
0.3
0.4
0.5
0.6
0.7
0.8
0.9
Dimension1(38.882%)
Group
CK
CRr
CRrP
CPT
Omics
Microbiome
Metabolome
